# COVID-19 and telehealth in the intensive care unit setting: a survey

**DOI:** 10.1186/s12913-022-08197-7

**Published:** 2022-06-20

**Authors:** Sarah E. Nelson, Jon Steuernagle, Leo Rotello, Paul Nyquist, Jose I. Suarez, Wendy Ziai

**Affiliations:** 1 Johns Hopkins University, 1800 Orleans St, Baltimore, MD 21287 USA; 2 Department of Neurosurgery and Neurology, Mount Sinai West, 1000 10th Avenue, New York, NY 10019 USA

**Keywords:** COVID-19, Telemedicine, Critical care, Health care surveys, Professional practice, Technology

## Abstract

**Background:**

Coronavirus disease (COVID-19) has led to changes in how healthcare is delivered. Here, through the administration of surveys, we evaluated telehealth use and views in US intensive care units (ICUs) during the pandemic.

**Methods:**

From June 2020 to July 2021, voluntary, electronic surveys were provided to ICU leaders of Johns Hopkins Medical Institution (JHMI) hospitals, members of the Neurocritical Care Society (NCS) who practice in the US, and Society of Critical Care Medicine (SCCM) members practicing adult medicine.

**Results:**

Response rates to our survey were as follows: 18 of 22 (81.8%) JHMI-based ICU leaders, 22 of 2218 (1.0%) NCS members practicing in the US, and 136 of 13,047 (1.0%) SCCM members. COVID-19 patients were among those cared for in the ICUs of 77.7, 86.4, and 93.4% of respondents, respectively, in April 2020 (defined as the peak of the pandemic). Telehealth technologies were used by 88.9, 77.3, and 75.6% of respondents, respectively, following the start of COVID-19 while only 22.2, 31.8, and 43.7% utilized them prior. The most common telehealth technologies were virtual meeting software and telephone (with no video component). Provider, nurse, and patient communications with the patient’s family constituted the most frequent types of interactions utilizing telehealth. Most common reasons for telehealth use included providing an update on a patient’s condition and conducting a goals of care discussion. 93.8–100.0% of respondents found telehealth technologies valuable in managing patients. Technical issues were noted by 66.7, 50.0, and 63.4% of respondents, respectively.

**Conclusions:**

Telehealth use increased greatly among respondents following the start of COVID-19. In US ICUs, telehealth technologies found diverse uses during the pandemic. Future studies are needed to confirm our findings.

**Supplementary Information:**

The online version contains supplementary material available at 10.1186/s12913-022-08197-7.

## Introduction

The pandemic caused by coronavirus disease 2019 (COVID-19), the infectious disease due to the novel coronavirus SARS-CoV-2, has caused drastic changes in medical institutions around the world. In addition to necessitating an increase in intensive care unit (ICU) capacity and personal protective equipment resources [1], new hospital policies were put into place, such as visitor restrictions [2] and the reduction of in-person outpatient appointments [3]. Implications of the latter two changes included modifications in how communication occurred among providers, patients, and families, and use of telemedicine increased significantly, particularly in the outpatient setting [4]. Fortunately, technology has come a long way over the past few decades: Devices such as smart phones and tablets as well as computer programs permitting audio-visual capabilities provide multiple possible ways to facilitate communication. Notably, Medicare and other insurance providers had begun covering for inpatient (including ICU) services during the pandemic making telehealth in the ICU even more feasible [5].

Use of telemedicine in the intensive care unit during the COVID-19 pandemic is important to study – especially since this is a setting in which provider-patient communication is often hindered due to intubation and/or severity of illness and where fast-paced changes in clinical status require streamlined communication among providers, families, and (to the extent possible) patients. While this use has been noted in the literature (for example, [6–10]) to our knowledge this is the first survey-based study on this topic that was intended to capture attitudes and uses of telehealth technologies by ICU providers on at least a national scale.

## Materials and methods

This was a 3-part study that included surveying (1) ICU leaders of the 5 major medical centers within the Johns Hopkins Medical Institution (JHMI; includes The Johns Hopkins Hospital, Johns Hopkins Bayview Medical Center, Howard County General Hospital, Sibley Memorial Hospital, and Suburban Hospital), (2) all US-based Neurocritical Care Society (NCS) members, and (3) members of the Society of Critical Care Medicine (SCCM) who practice adult medicine.

The survey was created in SurveyMonkey, titled “Survey on Telehealth Use in the ICU” (with the abbreviations NCS and SCCM added for the surveys distributed to each of these groups), and electronically distributed from June 2020 to July 2021. The survey instrument asked the following questions of participants: their professional role in the ICU, hospital at which they work (JHMI survey only), hospital setting in which they work (e.g., academic medical center, community hospital; NCS and SCCM surveys only), average daily patient census in their ICU, which patient populations their ICU cared for during the peak of the pandemic (defined as April 2020; answer choices included COVID-19 patients, non-COVID-19 patients, or both), whether their ICU used telehealth prior to the pandemic, whether their ICU has used telehealth technologies since the start of the pandemic, types of telehealth used since the start of the pandemic (e.g., telephone without video component, virtual meeting software, Facetime or WhatsApp), the types of interactions telehealth has been used for since the pandemic began (e.g., nurse to provider, nurse to patient’s family, patient to patient’s family), reasons telehealth has been used since the start of the pandemic (e.g., update on patient condition, consent for procedure, goals of care discussion), whether they experienced any technical issues, whether they feel telehealth technologies have been valuable in taking care of patients during the pandemic, whether they feel it will be useful to continue using telehealth technologies after hospital visitor restrictions are lifted, whether they feel telehealth technologies may create distrust among communicating parties due to not being able to communicate in person, and whether they would be interested in learning more about how telehealth could be used in their ICU.

A pilot survey was initially provided to 2 physicians (Drs. Steuernagle and Ziai), then following their feedback the survey was revised before first being sent to JHMI ICU leaders (June through July 2020) followed by NCS members (September through October 2020) and then to SCCM members (May through July 2021). Recruitment for the survey included posting on a centralized Johns Hopkins website and on the NCS website, sending emails to the leaders of JHMI ICUs informing them of the study, and an email sent to the target SCCM population. There was no other contact with participants.

Following return of the surveys, results were compiled for each part of the study (Supplemental Tables 1, 2 and 3). Statistics were computed by SurveyMonkey and included absolute numbers of responses per answer choice and frequency of answer choice. The response rate for each survey was also calculated (number of received surveys divided by number distributed).

## Results

Eighteen of 22 JHMI-based ICU leaders (81.8%) responded to the survey as did 22 of 2218 (1.0%) US-based NCS members and 136 of 13,047 (1.0%) SCCM members who practice adult medicine. The majority of respondents were medical/critical care directors (72.2%) in the JHMI survey; “other physicians” was the most frequent response in the NCS and SCCM surveys (45.5 and 37.5%, respectively). ICU leaders from all 5 hospitals within JHMI responded to their respective survey (Supplemental Table 1). The most common hospital setting was academic medical center in the NCS (77.3%; Supplemental Table 2) and SCCM (53.7%; Supplemental Table 3) surveys. Most SCCM respondents were located in the US (89.7%). In April 2020, defined in our survey as the peak of the pandemic, the ICUs of 77.7, 86.4, and 93.4% of respondents in the JHMI, NCS, and SCCM surveys were caring for COVID-19 patients, respectively.

**Fig. 1:**
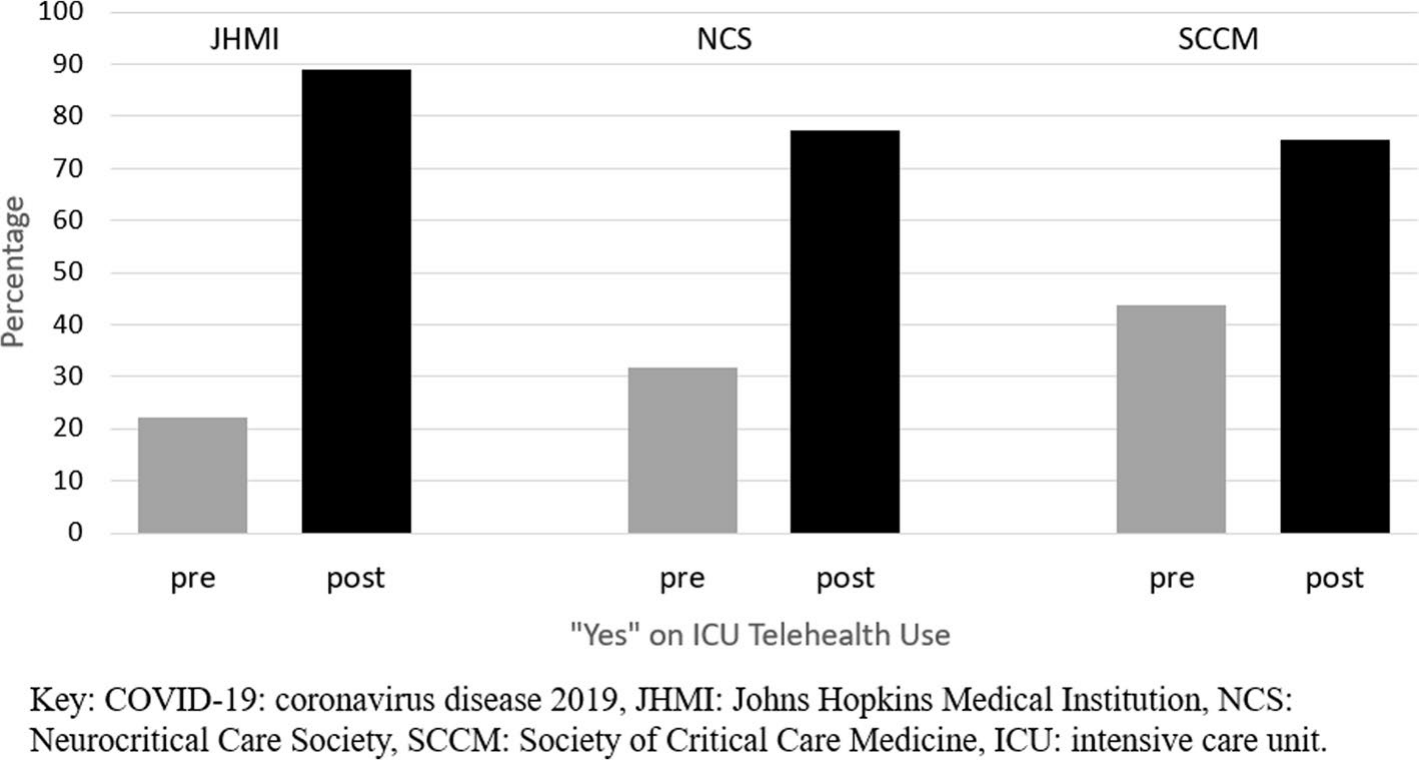
Percentages of “yes” responses to survey questions about having used telehealth prior to (“pre”) and after the start (“post”) of the COVID-19 pandemic in the JHMI, NCS, and SCCM Surveys

**Fig. 2:**
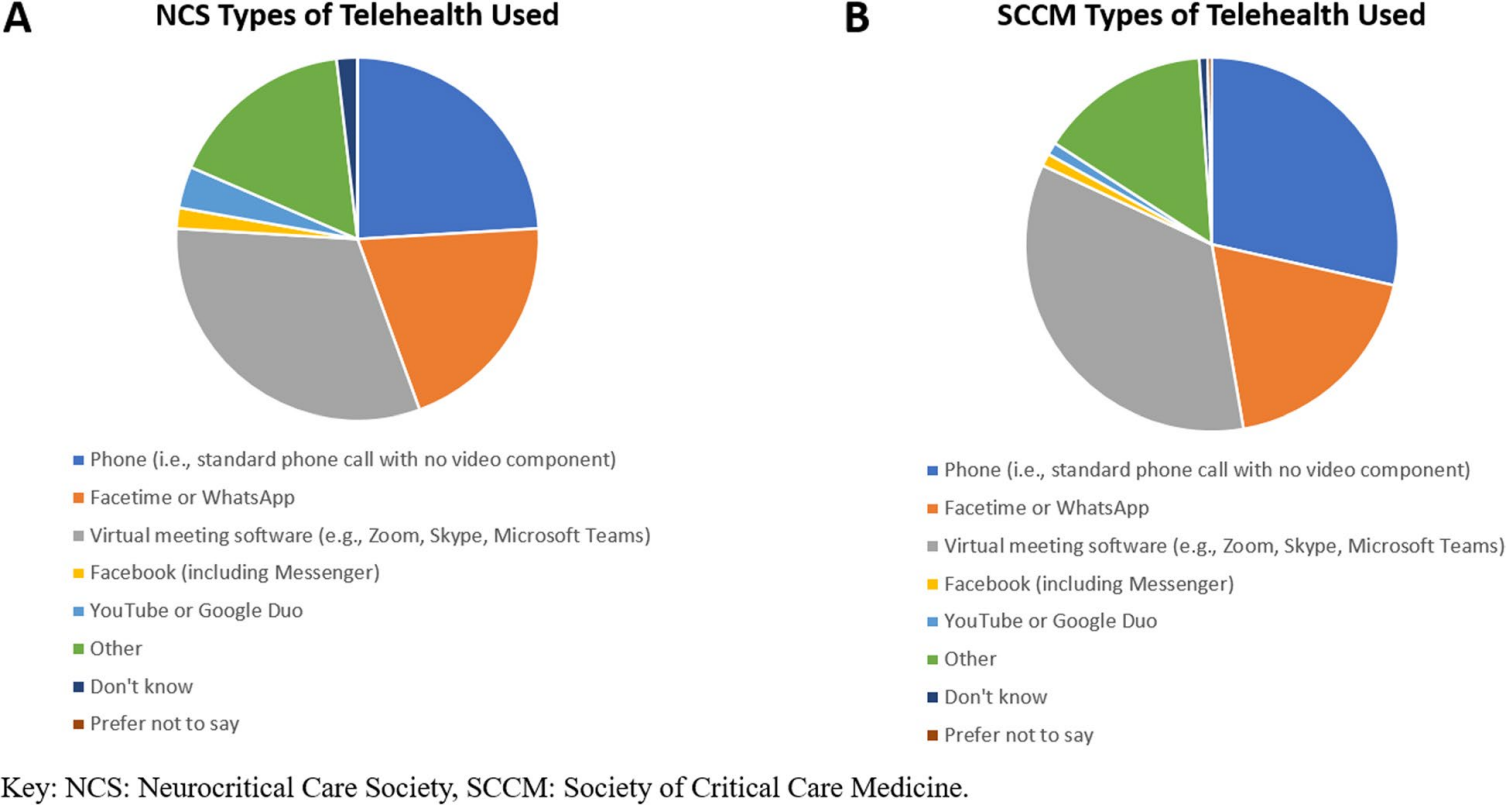
Types of telehealth used since the start of the COVID-19 pandemic among NCS (A) and SCCM (B) survey respondents

**Fig. 3:**
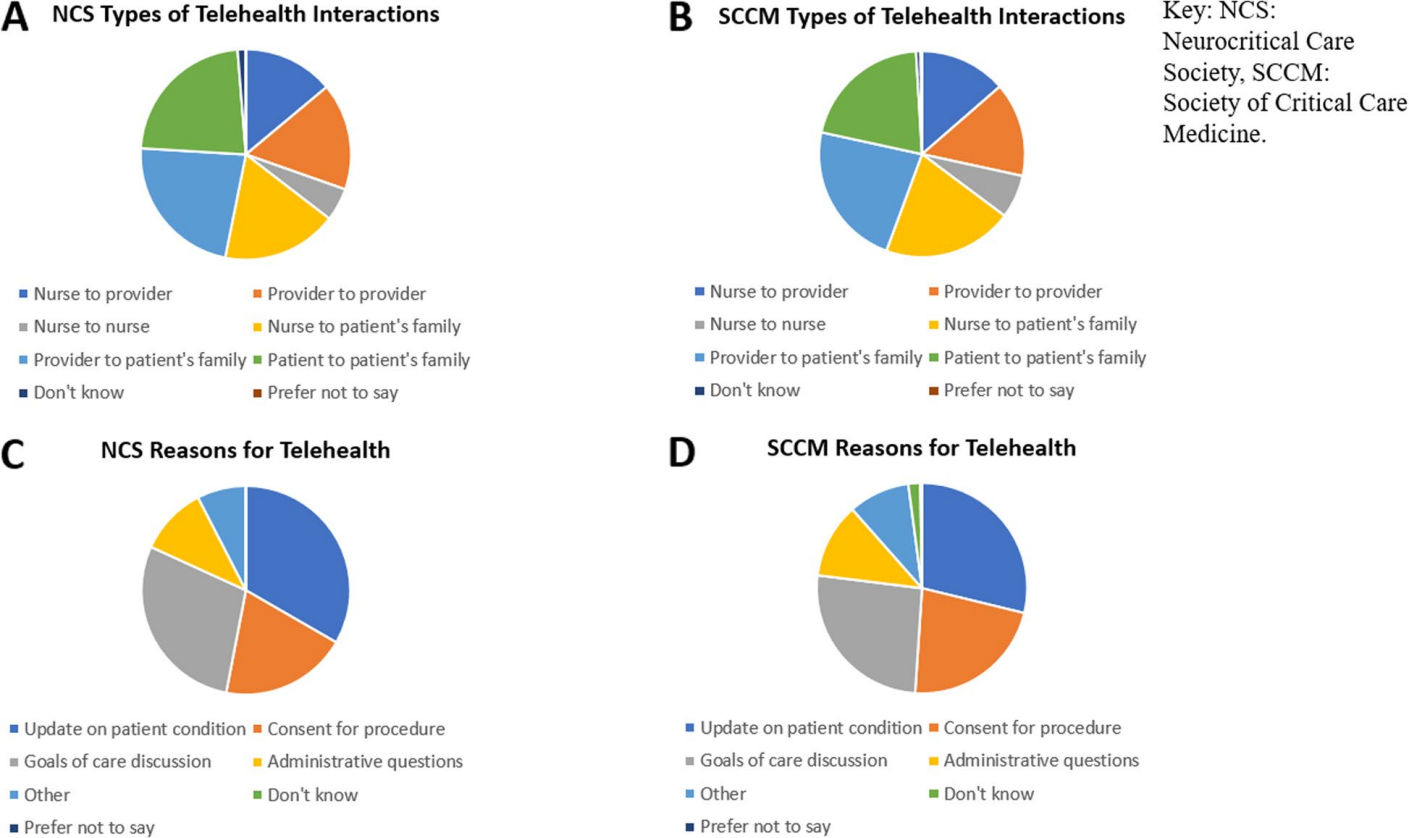
Types of telehealth interactions (**A**, **B**) and reasons for these interactions (**C**, **D**) since the start of the COVID-19 pandemic among NCS and SCCM survey respondents, respectively

While prior to the pandemic 22.2, 31.8, and 43.7% of respondents used telehealth, in the era of COVID-19 88.9, 77.3, and 75.6%, respectively, have used telehealth technologies (Fig. 1). A variety of telehealth technologies were used by respondents in all 3 surveys, but the most common were virtual meeting software followed by telephone (with no video component) (Fig. 2). Telehealth was used for a variety of types of interactions in the ICU, with the most common being provider, nurse, and patient interactions with the patient’s family (Fig. 3). Communications using telehealth also occurred for multiple reasons; most frequently these were to provide an update on a patient’s condition, conduct a goals of care discussion, and obtain consent for a procedure (Fig. 3).

Overall, telehealth technologies were seen as valuable in taking care of patients by 93.8 to 100.0% of respondents. 75.1, 93.8, and 89.0%, respectively, felt it would be useful for their ICU to continue using telehealth technologies after hospital visitor restrictions are lifted. Nonetheless, technical issues with telehealth technologies were experienced by 66.7, 50.0, and 63.4% of respondents, respectively. In addition, some respondents felt that telehealth may create mistrust due to not being able to communicate in person (25.0, 12.6, and 21.3%, respectively).

## Discussion

In this study, we found that response rates varied widely among the 3 surveys but that there were common themes: At the height of the pandemic the ICUs of most respondents included COVID-19 patients. In addition, telehealth was not used by the majority of respondents’ ICUs prior to COVID-19 but was utilized by most following the start of the pandemic. Categories of telehealth technologies, the types of interactions for which they were used, and the reasons for these interactions were generally consistent across all 3 surveys. Finally, while many experienced technical issues with telehealth and some had concerns regarding the potential to create mistrust by using telehealth, most respondents felt that these technologies were valuable in helping to manage patients and would be useful to continue utilizing in the future.

During the COVID-19 pandemic, use of telehealth expanded greatly. While a 2018 survey found that about 18% of physicians and < 10% of US residents had used telemedicine, this changed during the pandemic. Uptake was facilitated by providers being able to practice virtually in other states, improved reimbursement for telemedicine, audio-only visits being permitted, and Medicare and other insurers making telemedicine more financially accessible to patients [4, 11]. Approximately 30% of total outpatient visits for 16.7 million people with Medicare Advantage or commercial insurance were completed using telemedicine from March 18–June 16, 2020 [4]. A systematic review published early on in the pandemic found that telehealth improved the providing of health services, including both screening and monitoring of COVID-19 patients, conducting clinical research, and providing outpatient and inpatient services [12].

Use of telemedicine in the ICU setting during the COVID-19 pandemic has been investigated in some studies [6–10], and is important to examine due to hospital visitor restrictions, lack of capacity to communicate by many COVID-19 patients receiving mechanical ventilation and sedation [13, 14], and efforts by critical care teams to minimize exposure to COVID-19 [15]. In one of the more heartbreaking aspects of the pandemic, patients have unfortunately been left to die alone given the restrictions in place [13, 14], and non-COVID-19 inpatients also have suffered from lack of communication with their families given hospital visitor restrictions [16]. To our knowledge a US-wide evaluation on use of telemedicine in the ICU setting during the pandemic had not been conducted prior to our study but is crucial given a differential distribution of critical care resources such as beds and personnel across the US [17–19].

The results of our study suggest that telehealth can not only be implemented in many types of ICU settings in the US but further may be used to ameliorate some of the concerns discussed above. In fact, literature published prior to the COVID-19 pandemic demonstrated that telemedicine in the ICU can lead to improved access to care, reduced length of stay, better ability to follow clinical guidelines, improved communication, educational opportunities, and likely cost savings [20–22]. While our study did not specifically investigate financial and patient care-related outcomes, our results seem to confirm that telehealth may assist access to care and facilitate communication with families.

Though our experience during the pandemic now suggests that telemedicine can be an important adjunct to medical care, it is also apparent that with the expiration of pandemic policies that permitted this telemedicine expansion there will need to be further efforts to ensure continued access to telemedicine for patients [11]. While ICU care conducted via telehealth continues to be at least partly covered by Medicare [5] there remains uncertainty with regards to how long this coverage will continue. For instance, two bills have recently been under discussion by the US Congress that would extend some of these flexibilities regarding telehealth that were introduced during the COVID-19 pandemic and help ensure the availability and feasibility of this communication modality to Americans [23, 24].

Limitations of the study include the fact that response rates to the NCS and SCCM surveys were low despite broad advertising thus subjecting results to selection bias. In addition, the survey was not approved by SCCM for several months thus the notations regarding mid-March and April in the survey may have been ambiguous as to year to some respondents though phrases such as “peak of the pandemic” and “Since the start of the COVID-19 pandemic” were used that likely helped make this distinction. Further, we did not investigate the relationship between telehealth and patient outcome in ICU patients as well as costs. In addition, while we did attempt to collect types and reasons for telehealth (which also included uses by clinicians to manage patients), nonetheless we did not comprehensively investigate these aspects of telehealth in order to keep the survey short. Nonetheless, a major strength of our study is the large population to which the survey was sent and the fact that this is one of the only studies (to our knowledge) that investigates use of telehealth in the ICU setting across the US during the COVID-19 pandemic.

## Conclusions

While response rates were lower for the NCS and SCCM surveys as compared to the JHMI-based survey, common trends of our broadly administered survey were that most ICUs cared for COVID-19 patients and most did not utilize telehealth prior to COVID-19 while the vast majority did following the start of the pandemic. These technologies were generally seen as valuable and likely useful for the future. Telehealth may be an important tool in facilitating ICU care moving forward. Additional studies are needed to confirm and expand upon our findings as well as to evaluate the relationship between use of telehealth technologies in the ICU setting and outcomes including hospital costs.

## Supplementary Information

**Additional file 1.**

## Data Availability

Data is available upon reasonable request from the corresponding author but can also be found in the supplementary data.

## Abbreviations

COVID-19: Coronavirus disease 2019
JHMI: Johns Hopkins Medical Institution
NCS: Neurocritical Care Society
SCCM: Society of Critical Care Medicine

## References

[1] Phua J, Weng L, Ling L (2020). Intensive care management of coronavirus disease 2019 (COVID-19): challenges and recommendations. Lancet Respir Med.

[2] Centers for Disease Control and Prevention. Management of Visitors to Healthcare Facilities in the Context of COVID-19: Non-US Healthcare Settings, https://www.cdc.gov/coronavirus/2019-ncov/hcp/non-us-settings/hcf-visitors.html; 2020 [Accessed 30 Sep 2021].

[3] Mehrotra A, Chernew M, Linetsky D, et al. The Impact of COVID-19 on Outpatient Visits in 2020: Visits Remained Stable, Despite a Late Surge in Cases, https://www.commonwealthfund.org/publications/2021/feb/impact-covid-19-outpatient-visits-2020-visits-stable-despite-late-surge; 2021 [Accessed 28 November 2021].

[4] Patel BSY, Mehrotra A, Huskamp HA (2021). Variation in telemedicine use and outpatient care during the COVID-19 pandemic in the United States. Health Aff.

[5] Centers for Medicare & Medicaid Services. MLN Fact Sheet: Telehealth Services, https://www.cms.gov/Outreach-and-Education/Medicare-Learning-Network-MLN/MLNProducts/Downloads/TelehealthSrvcsfctsht.pdf; 2021 [Accessed 30 Sep 2021].

[6] Ieronimakis KM, Cain JA, Switzer MS (2020). Leveraging tele-critical care capabilities for clinical trial consent. Crit Care Explor.

[7] Kennedy NR, Steinberg A, Arnold RM (2021). Perspectives on telephone and video communication in the intensive care unit during COVID-19. Ann Am Thorac Soc.

[8] Krouss M, Allison MG, Rios S (2020). Rapid implementation of Telecritical care support during a pandemic: lessons learned during the coronavirus disease 2020 surge in new York City. Crit Care Explor..

[9] Negro A, Mucci M, Beccaria P (2020). Introducing the video call to facilitate the communication between health care providers and families of patients in the intensive care unit during COVID-19 pandemia. Intensive Crit Care Nurs.

[10] Sasangohar F, Dhala A, Zheng F (2021). Use of telecritical care for family visitation to ICU during the COVID-19 pandemic: an interview study and sentiment analysis. BMJ Qual Saf.

[11] Volk J, Palanker D, O’Brien M, et al. States’ Actions to Expand Telemedicine Access During COVID-19 and Future Policy Considerations, https://www.commonwealthfund.org/publications/issue-briefs/2021/jun/states-actions-expand-telemedicine-access-covid-19; 2021 [Accessed 30 Sep 2021]. 10.26099/r95z-bs17.

[12] Monaghesh E, Hajizadeh A (2020). The role of telehealth during COVID-19 outbreak: a systematic review based on current evidence. BMC Public Health.

[13] Wakam GK, Montgomery JR, Biesterveld BE (2020). Not dying alone - modern compassionate Care in the Covid-19 pandemic. N Engl J Med.

[14] Zapana V. Texts from my Father, in Elmhurst Hospital, https://www.newyorker.com/magazine/2020/04/13/texts-from-my-father-in-elmhurst-hospital; 2020 [Accessed 30 Sep 2021].

[15] Centers for Disease Control and Prevention. Using Telehealth to Expand Access to Essential Health Services during the COVID-19 Pandemic, https://www.cdc.gov/coronavirus/2019-ncov/hcp/telehealth.html; 2020 [Accessed 30 Sep 2021].

[16] Lamas DJ. I’m on the Front Lines. I Have No Plan for This, https://www.nytimes.com/2020/03/24/opinion/coronavirus-hospital-visits.html; 2020 [Accessed 30 Sep 2021].

[17] Merkel MJ, Edwards R, Ness J (2020). Statewide real-time tracking of beds and ventilators during coronavirus disease 2019 and beyond. Crit Care Explor..

[18] Lopez E. The Critical Care Workforce and COVID-19: A State-by-State Analysis, https://www.kff.org/report-section/the-critical-care-workforce-and-covid-19-a-state-by-state-analysis-table/; 2020 [Accessed 10 Jan 2022].

[19] Halpern N, Tan K. United States Resource Availability for COVID-19, https://www.sccm.org/Blog/March-2020/United-States-Resource-Availability-for-COVID-19; 2020 [Accessed 10 Jan 2022].

[20] Romig MC, Latif A, Gill RS (2012). Perceived benefit of a telemedicine consultative service in a highly staffed intensive care unit. J Crit Care.

[21] Becker CD, Fusaro MV, Scurlock C (2019). Telemedicine in the ICU: clinical outcomes, economic aspects, and trainee education. Curr Opin Anaesthesiol.

[22] Avdalovic MV, Marcin JP (2019). When will telemedicine appear in the ICU?. J Intensive Care Med.

[23] 117th Congress. H.R.2903 - CONNECT for Health Act of 2021, https://www.congress.gov/bill/117th-congress/house-bill/2903?s=1&r=4; 2021 [Accessed 30 Sep 2021].

[24] 117th Congress. H.R.1332 - Telehealth Modernization Act, https://www.congress.gov/bill/117th-congress/house-bill/1332; 2021 [Accessed 30 Sep 2021].

