# Supplementary material for: COVID-19 and telehealth in the intensive care unit setting: a survey
Source: BMC Health Serv Res. 2022 Jun 20;22:797. doi: 10.1186/s12913-022-08197-7 (PMC9208537; doi:10.1186/s12913-022-08197-7)
Supplement: Supplementary file 1 — Additional file 1. [file 12913_2022_8197_MOESM1_ESM.docx]

**SUPPLEMENTAL TABLES**

**Supplemental Table 1. Survey Questions Administered to Johns Hopkins Medical Institution physicians and Responses Provided**

| *Question* | *Answer Choices* | *Responses* |
| --- | --- | --- |
| What is your professional role in the ICU? | 1. Medical director or Critical care director 2. Nurse manager 3. Charge nurse 4. Other physician 5. Other professional role 6. Prefer not to say | 1. 13 (72.2%) 2. 3 (16.7%) 3. 0 (0%) 4. 1 (5.6%) 5. 1 (5.6%) 6. 0 (0%) |
| At which hospital is the ICU referred to in question 1? | 1. Howard County General Hospital 2. Johns Hopkins Hospital 3. Bayview Medical Center 4. Sibley Memorial Hospital 5. Suburban Hospital 6. Prefer not to say | 1. 4 (22.2%) 2. 4 (22.2%) 3. 4 (22.2%) 4. 1 (5.6%) 5. 5 (27.8%) 6. 0 (0%) |
| On average, what is the daily patient census in your ICU? | 1. <10 2. 11-20 3. 21-30 4. >30 5. Don’t know 6. Prefer not to say | 1. 2 (11.1%) 2. 10 (55.6%) 3. 5 (27.8%) 4. 0 (0%) 5. 0 (0%) 6. 1 (5.6%) |
| During the peak of the pandemic in April 2020, your ICU cared for which of the following patient populations? | 1. COVID-19 patients only 2. Non-COVID-19 patients 3. Both COVID-19 ad non-COVID-19 patients 4. Don’t know 5. Prefer not to say | 1. 6 (33.3%) 2. 4 (22.2%) 3. 8 (44.4%) 4. 0 (0%) 5. 0 (0%) |
| Prior to the start of the COVID-19 pandemic, did your ICU use telehealth (e.g., phone, virtual meeting software, other video communication)? | 1. Yes 2. No 3. Don’t know 4. Prefer not to say | 1. 4 (22.2%) 2. 14 (77.8%) 3. 0 (0%) 4. 0 (0%) |
| Since the start of the COVID-19 pandemic in mid-March, has your ICU utilized new telehealth technologies (e.g., phone, virtual meeting software, other video communication)? | 1. Yes 2. No 3. Don’t know 4. Prefer not to say | 1. 16 (88.9%) 2. 2 (11.1%) 3. 0 (0%) 4. 0 (0%) |
| Since the start of the COVID-19 pandemic in mid-March, what types of telehealth has your ICU utilized? Select all that apply. | 1. Phone (i.e., standard phone call with no video component) 2. Facetime or WhatsApp 3. Virtual meeting software (e.g., Zoom, Skype, Microsoft Teams) 4. Facebook (including Messenger) 5. YouTube or Google Duo 6. Other 7. Don’t know 8. Prefer not to say | 1. 12 (66.7%) 2. 3 (16.7%) 3. 18 (100.0%) 4. 1 (5.6%) 5. 0 (0%) 6. 3 (16.7%) 7. 0 (0%) 8. 0 (0%) 9. 0 (0%) 10. 0 (0%) |
| Since the start of the COVID-19 pandemic in mid-March, what type(s) of interactions has telehealth been utilized for in your ICU? Select all that apply. | 1. Nurse to provider 2. Provider to provider 3. Nurse to nurse 4. Nurse to patient’s family 5. Provider to patient’s family 6. Patient to patient’s family 7. Don’t know 8. Prefer not to say | 1. 5 (27.8%) 2. 8 (44.4%) 3. 5 (27.8%) 4. 16 (88.9%) 5. 15 (83.3%) 6. 15 (83.3%) 7. 0 (0%) 8. 0 (0%) |
| What are reasons telehealth has been used in your ICU since the start of the COVID-19 pandemic? Select all that apply. | 1. Update on patient condition 2. Consent for procedure 3. Goals of care discussion 4. Administrative questions 5. Other 6. Don’t know 7. Prefer not to say | 1. 16 (88.9%) 2. 13 (72.2%) 3. 16 (88.9%) 4. 9 (50.0%) 5. 5 (27.8%) 6. 0 (0%) 7. 0 (0%) |
| Did you experience any technical issues with using telehealth since the start of the COVID-19 pandemic? | 1. Yes 2. No 3. Don’t know 4. Prefer not to say | 1. 12 (66.7%) 2. 6 (33.3%) 3. 0 (0%) 4. 0 (0%) |
| Telehealth technologies have been VALUABLE in taking care of patients during the COVID-19 pandemic. | 1. Strongly agree 2. Agree 3. Neither agree nor disagree 4. Disagree 5. Strongly disagree | 1. 9 (56.3%) 2. 6 (37.5%) 3. 0 (0%) 4. 1 (6.3%) 5. 0 (0%) |
| I feel that it will be useful for my ICU to continue to utilize telehealth technologies AFTER hospital visitor restrictions have been lifted. | 1. Strongly agree 2. Agree 3. Neither agree nor disagree 4. Disagree 5. Strongly disagree | 1. 7 (43.8%) 2. 5 (31.3%) 3. 3 (18.8%) 4. 0 (0%) 5. 1 (6.3%) |
| I feel that telehealth technologies may CREATE MISTRUST among communicating parties due to not being able to communicate in person. | 1. Strongly agree 2. Agree 3. Neither agree nor disagree 4. Disagree 5. Strongly disagree | 1. 2 (12.5%) 2. 2 (12.5%) 3. 1 (6.3%) 4. 7 (43.8%) 5. 4 (25.0%) |
| I would be interested in learning more about how telehealth could be used in my ICU. | 1. Yes 2. No 3. Don’t know 4. Prefer not to say | 1. 14 (77.8%) 2. 2 (11.1%) 3. 2 (11.1%) 4. 0 (0%) |

**Supplemental Table 2. Survey Questions Administered to the Neurocritical Care Society and Responses Provided**

| *Question* | *Answer Choices* | *Responses* |
| --- | --- | --- |
| What is your professional role in the ICU? | 1. Medical director or Critical care director 2. Nurse manager or Charge nurse 3. Advanced practice provider 4. Pharmacist 5. Medical student, Resident, or Fellow 6. Other physician 7. Other professional role 8. Prefer not to say | 1. 7 (31.8%) 2. 0 (0%) 3. 3 (13.6%) 4. 1 (4.6%) 5. 0 (0%) 6. 10 (45.5%) 7. 1 (4.6%) 8. 0 (0%) |
| Which best describes the hospital setting in which you work? | 1. Academic medical center 2. Private medical center/hospital 3. Community hospital 4. Solo practice 5. Other 6. Prefer not to say | 1. 17 (77.3%) 2. 3 (13.6%) 3. 0 (0%) 4. 2 (9.1%) 5. 0 (0%) 6. 0 (0%) 7. 0 (0%) |
| On average, what is the daily patient census in your ICU? | 1. <10 2. 11-20 3. 21-30 4. >30 5. Don’t know 6. Prefer not to say | 1. 0 (0%) 2. 12 (54.6%) 3. 4 (18.2%) 4. 4 (18.2%) 5. 2 (9.1%) 6. 0 (0%) |
| During the peak of the pandemic in April 2020, your ICU cared for which of the following patient populations? | 1. COVID-19 patients only 2. Non-COVID-19 patients 3. Both COVID-19 and non-COVID-19 patients 4. Don’t know 5. Prefer not to say | 1. 2 (9.1%) 2. 2 (13.6%) 3. 17 (77.3%) 4. 0 (0%) 5. 0 (0%) |
| Prior to the start of the COVID-19 pandemic, did your ICU use telehealth (e.g., phone, virtual meeting software, other video communication)? | 1. Yes 2. No 3. Don’t know 4. Prefer not to say | 1. 7 (31.8%) 2. 15 (68.2%) 3. 0 (0%) 4. 0 (0%) |
| Since the start of the COVID-19 pandemic in mid-March, has your ICU utilized new telehealth technologies (e.g., phone, virtual meeting software, other video communication)? | 1. Yes 2. No 3. Don’t know 4. Prefer not to say | 1. 17 (77.3%) 2. 3 (13.6%) 3. 2 (9.1%) 4. 0 (0%) |
| Since the start of the COVID-19 pandemic in mid-March, what types of telehealth has your ICU utilized? Select all that apply. | 1. Phone (i.e., standard phone call with no video component) 2. Facetime or WhatsApp 3. Virtual meeting software (e.g., Zoom, Skype, Microsoft Teams) 4. Facebook (including Messenger) 5. YouTube or Google Duo 6. Other 7. Don’t know 8. Prefer not to say | 1. 13 (59.1%) 2. 11 (50.0%) 3. 17 (77.3%) 4. 1 (4.6%) 5. 2 (9.1%) 6. 9 (40.9%) 7. 1 (4.6%) 8. 0 (0%) 9. 0 (0%) 10. 0 (0%) |
| Since the start of the COVID-19 pandemic in mid-March, what type(s) of interactions has telehealth been utilized for in your ICU? Select all that apply. | 1. Nurse to provider 2. Provider to provider 3. Nurse to nurse 4. Nurse to patient’s family 5. Provider to patient’s family 6. Patient to patient’s family 7. Don’t know 8. Prefer not to say | 1. 11 (50.0%) 2. 13 (59.1%) 3. 4 (18.2%) 4. 14 (63.6%) 5. 18 (81.8%) 6. 18 (81.8%) 7. 1 (4.6%) 8. 0 (0%) |
| What are reasons telehealth has been used in your ICU since the start of the COVID-19 pandemic? Select all that apply. | 1. Update on patient condition 2. Consent for procedure 3. Goals of care discussion 4. Administrative questions 5. Other 6. Don’t know 7. Prefer not to say | 1. 22 (100.0%) 2. 13 (59.1%) 3. 19 (86.4%) 4. 7 (31.8%) 5. 5 (22.7%) 6. 0 (0%) 7. 0 (0%) |
| Did you experience any technical issues with using telehealth since the start of the COVID-19 pandemic? | 1. Yes 2. No 3. Don’t know 4. Prefer not to say | 1. 11 (50.0%) 2. 7 (31.8%) 3. 4 (18.2%) 4. 0 (0%) |
| Telehealth technologies have been VALUABLE in taking care of patients during the COVID-19 pandemic. | 1. Strongly agree 2. Agree 3. Neither agree nor disagree 4. Disagree 5. Strongly disagree | 1. 8 (50.0%) 2. 8 (50.0%) 3. 0 (0%) 4. 0 (0%) 5. 0 (0%) |
| I feel that it will be useful for my ICU to continue to utilize telehealth technologies AFTER hospital visitor restrictions have been lifted. | 1. Strongly agree 2. Agree 3. Neither agree nor disagree 4. Disagree 5. Strongly disagree | 1. 7 (43.8%) 2. 8 (50.0%) 3. 1 (6.3%) 4. 0 (0%) 5. 0 (0%) |
| I feel that telehealth technologies may CREATE MISTRUST among communicating parties due to not being able to communicate in person. | 1. Strongly agree 2. Agree 3. Neither agree nor disagree 4. Disagree 5. Strongly disagree | 1. 1 (6.3%) 2. 1 (6.3%) 3. 5 (31.3%) 4. 7 (43.8%) 5. 2 (12.5%) |
| I would be interested in learning more about how telehealth could be used in my ICU. | 1. Yes 2. No 3. Don’t know 4. Prefer not to say | 1. 11 (52.4%) 2. 5 (23.8%) 3. 3 (14.3%) 4. 2 (9.5%) |

**Supplemental Table 3. Survey Questions Administered to the Society of Critical Care Medicine and Responses Provided**

| *Question* | *Answer Choices* | *Responses* |
| --- | --- | --- |
| What is your professional role in the ICU? | 1. Medical director or Critical care director 2. Nurse manager or Charge nurse 3. Advanced practice provider 4. Pharmacist 5. Medical student, Resident, or Fellow 6. Other physician 7. Other professional role 8. Prefer not to say | 1. 42 (30.9%) 2. 3 (2.2%) 3. 16 (11.8%) 4. 11 (8.1%) 5. 3 (2.2%) 6. 51 (37.5%) 7. 10 (7.4%) 8. 0 (0%) |
| Which best describes the hospital setting in which you work? | 1. Academic medical center 2. Private medical center/hospital 3. Government-based hospital 4. Community hospital 5. Solo practice 6. Other 7. Prefer not to say | 1. 73 (53.7%) 2. 23 (16.9%) 3. 3 (2.2%) 4. 34 (25.0%) 5. 0 (0%) 6. 3 (2.2%) 7. 0 (0%) |
| In what country do you work? | 1. United States 2. Internationally 3. Prefer not to say | 1. 122 (89.7%) 2. 14 (10.3%) 3. 0 (0%) |
| On average, what is the daily patient census in your ICU? | 1. <10 2. 11-20 3. 21-30 4. >30 5. Don’t know 6. Prefer not to say | 1. 17 (12.5%) 2. 55 (40.4%) 3. 26 (19.1%) 4. 35 (25.7%) 5. 1 (0.7%) 6. 2 (1.5%) |
| During the peak of the pandemic in April 2020, your ICU cared for which of the following patient populations? | 1. COVID-19 patients only 2. Non-COVID-19 patients 3. Both COVID-19 and non-COVID-19 patients 4. Don’t know 5. Prefer not to say | 1. 14 (10.4%) 2. 9 (6.7%) 3. 112 (83.0%) 4. 0 (0%) 5. 0 (0%) |
| Prior to the start of the COVID-19 pandemic, did your ICU use telehealth (e.g., phone, virtual meeting software, other video communication)? | 1. Yes 2. No 3. Don’t know 4. Prefer not to say | 1. 59 (43.7%) 2. 73 (54.1%) 3. 3 (2.2%) 4. 0 (0%) |
| Since the start of the COVID-19 pandemic in mid-March, has your ICU utilized new telehealth technologies (e.g., phone, virtual meeting software, other video communication)? | 1. Yes 2. No 3. Don’t know 4. Prefer not to say | 1. 102 (75.6%) 2. 32 (23.7%) 3. 1 (0.7%) 4. 0 (0%) |
| Since the start of the COVID-19 pandemic in mid-March, what types of telehealth has your ICU utilized? Select all that apply. | 1. Phone (i.e., standard phone call with no video component) 2. Facetime or WhatsApp 3. Virtual meeting software (e.g., Zoom, Skype, Microsoft Teams) 4. Facebook (including Messenger) 5. YouTube or Google Duo 6. Other 7. Don’t know 8. Prefer not to say | 1. 79 (59.9%) 2. 52 (39.4%) 3. 96 (72.7%) 4. 3 (2.3%) 5. 3 (2.3%) 6. 41 (31.1%) 7. 2 (1.5%) 8. 1 (0.8%) 9. 0 (0%) 10. 0 (0%) |
| Since the start of the COVID-19 pandemic in mid-March, what type(s) of interactions has telehealth been utilized for in your ICU? Select all that apply. | 1. Nurse to provider 2. Provider to provider 3. Nurse to nurse 4. Nurse to patient’s family 5. Provider to patient’s family 6. Patient to patient’s family 7. Don’t know 8. Prefer not to say | 1. 58 (43.9%) 2. 63 (47.7%) 3. 29 (22.0%) 4. 87 (65.9%) 5. 97 (73.5%) 6. 88 (66.7%) 7. 3 (2.3%) 8. 1 (0.8%) |
| What are reasons telehealth has been used in your ICU since the start of the COVID-19 pandemic? Select all that apply. | 1. Update on patient condition 2. Consent for procedure 3. Goals of care discussion 4. Administrative questions 5. Other 6. Don’t know 7. Prefer not to say | 1. 110 (82.7%) 2. 85 (63.9%) 3. 99 (74.4%) 4. 44 (33.1%) 5. 36 (27.1%) 6. 7 (5.3%) 7. 1 (0.8%) |
| Did you experience any technical issues with using telehealth since the start of the COVID-19 pandemic? | 1. Yes 2. No 3. Don’t know 4. Prefer not to say | 1. 85 (63.4%) 2. 37 (27.6%) 3. 11 (8.2%) 4. 1 (0.8%) |
| Telehealth technologies have been VALUABLE in taking care of patients during the COVID-19 pandemic. | 1. Strongly agree 2. Agree 3. Neither agree nor disagree 4. Disagree 5. Strongly disagree | 1. 63 (63.0%) 2. 34 (34.0%) 3. 1 (1.0%) 4. 1 (1.0%) 5. 1 (1.0%) |
| I feel that it will be useful for my ICU to continue to utilize telehealth technologies AFTER hospital visitor restrictions have been lifted. | 1. Strongly agree 2. Agree 3. Neither agree nor disagree 4. Disagree 5. Strongly disagree | 1. 54 (54.0%) 2. 35 (35.0%) 3. 7 (7.0%) 4. 2 (2.0%) 5. 2 (2.0%) |
| I feel that telehealth technologies may CREATE MISTRUST among communicating parties due to not being able to communicate in person. | 1. Strongly agree 2. Agree 3. Neither agree nor disagree 4. Disagree 5. Strongly disagree | 1. 5 (5.1%) 2. 16 (16.2%) 3. 26 (26.3%) 4. 40 (40.4%) 5. 12 (12.1%) |
| I would be interested in learning more about how telehealth could be used in my ICU. | 1. Yes 2. No 3. Don’t know 4. Prefer not to say | 1. 65 (48.9%) 2. 50 (37.6%) 3. 13 (9.8%) 4. 5 (3.8%) |
